# Effects of Secretoneurin and Gonadotropin-Releasing Hormone Agonist on the Spawning of Captive Greater Amberjack (*Seriola dumerili*)

**DOI:** 10.3390/life12091457

**Published:** 2022-09-19

**Authors:** Yanlong Song, Yinjun Jiang, Ji Chen, Binbin Tao, Wen Xu, Yang Huang, Guangli Li, Chunhua Zhu, Wei Hu

**Affiliations:** 1 Southern Marine Science and Engineering Guangdong Laboratory (Zhanjiang), Zhanjiang 524013, China; 2 Guangdong Laboratory for Lingnan Modem Agriculture, Guangzhou 510642, China; 3 State Key Laboratory of Freshwater Ecology and Biotechnology, Institute of Hydrobiology, Innovation Academy for Seed Design, Chinese Academy of Sciences, Wuhan 430072, China; 4 College of Life Sciences, University of Chinese Academy of Sciences, Beijing 100049, China

**Keywords:** secretoneurin, gonadotropin-releasing hormone, agonist, spawn, cultured greater amberjack

## Abstract

The greater amberjack (*Seriola dumerili*), a pelagic marine species with a global distribution, has considerable worldwide potential as an aquaculture species. However, difficulties have been encountered in inducing spontaneous spawning in cultured fish stocks. In this study, we analysed the key regulatory factors, secretoneurin (SN) and gonadotropin-releasing hormone (GnRH), in greater amberjack. Active peptides of SN and GnRH, SdSNa, and SdGnRH, respectively, were obtained by comparative analysis of homologous proteins from different species. Amino acid substitutions of the SdGnRH decapeptide at position 6 with a dextrorotatory (D) amino acid and at position 10 with an ethylamide group yielded a super-active agonist (SdGnRHa). The injection of SdSNa and SdGnRHa elevated luteinizing hormone, thyroid-stimulating hormone, and oxytocin levels in the sera of sexually mature fish, whereas it reduced the level of follicle-stimulating hormone. Furthermore, in response to the SdSNa and SdGnRHa injections, we detected an increase in the expression of genes associated with oocyte development and spermatogenesis. We established that the greater amberjack cultured along the southern coast of China reached sexual maturity at three years of age, and its reproductive season extended from February to April. Spawning of the cultured greater amberjack was successfully induced with a single injection of SdGnRHa/SdSN/DOM/HCG. Our findings indicate that similar to GnRHa, SNa is a potential stimulator of reproduction that can be used to artificially induce spawning in marine fish.

## 1. Introduction

The greater amberjack (*Seriola dumerili*, Risso 1810) is a large coastal epibenthic and pelagic teleost fish and is considered a potential aquaculture species worldwide. Although the greater amberjack is reported to adapt readily to a captive environment, obtaining reproductively mature fish and spontaneous spawning have been particularly challenging in cultured stocks [1]. The fertilisation and hatching of eggs spawned naturally by greater amberjack in captivity is commonly reported as low effective as 16.49% [2]. The inability to control reproduction in captivity has hindered the commercialisation of this species [3,4]. It is assumed that the lack of vitellogenesis, oocyte maturation (OM), and ovulation or spawning that is often observed in cultured fish is attributable to the absence of appropriate environmental stimuli and/or the effects of stressors imposed by conditions associated with a captive environment [5]. Although most captive female fish undergo vitellogenesis during the reproductive period, they fail to undergo OM and, consequently, fail to ovulate [6,7,8,9].

The mature gametes and subsequent spawning of fish are controlled by multiple neurohormonal factors in the brain and pituitary glands. The gonadotropin-releasing hormone (GnRH) decapeptide has long been considered the primary factor that regulates the secretion of gonadotropin hormones, which triggers a hormonal cascade that contributes to the control of reproduction via the hypothalamus-pituitary-gonad axis [10]. To date, three types of GnRH have been identified in teleosts. Most species express two types of GnRH, and some express all three. GnRH1 is expressed in specific neurons of the hypophysiotropic preoptic region and is associated with hypophysiotropic functions [10,11]. GnRH2 is found in most teleosts and is constitutively expressed in the synencephalon and mesencephalon. It has been shown to play a role in controlling satiation in zebrafish and maintaining optimal oocyte quality in female fish [12]. GnRH3 is expressed primarily in the ventromedial olfactory bulbs and terminal nerves [13] and is hypothesised to be involved in a range of neuromodulatory activities throughout the brain. Several hormonal treatments have previously been employed to induce OM, ovulation, and spawning in fish. Among these is the administration of GnRH agonists (GnRHas), which have been used instead of native peptides, as native GnRHs are known to be rapidly degraded in circulation by endopeptidases produced in the pituitary gland, liver, and kidney [14]. In addition to increased resistance to enzymatic cleavage, the modified GnRHa produced has a higher receptor binding affinity and is more potent than native GnRHs in inducing luteinizing hormone (LH) release [15,16,17]. In fish, the basal release of LH in the pituitary gland is typically inhibited by dopamine [18], and dopamine antagonists (DAs) have often been used in combination with GnRHa injections to eliminate the inhibition of gonadotropins and enhance the stimulatory activity of hormonal treatments [19].

Secretogranin-2 (SCG2) is a secretory protein distributed in the dense-core vesicles of multiple neurons and endocrine cells and serves as a precursor of secretoneurin (SN), produced via the activity of prohormone convertase in secretory granules [20]. Pioneering research in goldfish has indicated that SN is a regulator involved in the control of gonadotropin release and reproductive activities [21]. Moreover, these findings of in vitro studies have established that SN can directly stimulate LH production and release from dispersed goldfish pituitary cells [22,23]. Synthetic mouse SN has also been demonstrated to stimulate LH release from the mouse LβT gonadotroph cell line, which expresses LHβ and α-subunit mRNAs and responds to GnRH administration with LH secretion [24]. These observations indicate that SN is a conserved peptide that contributes to the regulation of gonadotropins in vertebrates. Teleosts express two *scg2* paralogs, *scg2a* and *scg2b*, which are assumed to be a consequence of whole-genome duplication that occurred in the teleost lineage [25]. Mutations in *scg2a* and *scg2b* disrupt sexual behaviour and spawning success in zebrafish. Furthermore, the expression of *gnrh3* in the hypothalamus and *lhβ* and glycoprotein hormones, alpha polypeptide (*cga*) in the pituitary gland, are significantly reduced in *scg2a* and *scg2b* double-mutant fish [26]. Consequently, it can be speculated that the SN may play an important role in regulating reproductive behaviour and the synthesis and release of gonadotropin in fish.

To address the long-standing problem of gamete maturation and spawning of cultured greater amberjack, we initially analysed the native GnRH and SN of this species and subsequently synthesised a specific GnRH analogue of *S. dumerili* (SdGnRHa) and secretoneurin core peptide (SdSN). Along with a dopamine receptor antagonist, we used these synthetic peptides as the main components to produce a specific hormone cocktail, which was found to have a pronounced stimulatory effect on promoting pituitary hormone release and examined its effects on greater amberjack reproduction.

## 2. Materials and Methods

### 2.1. Broodstock Maintenance

Juvenile greater amberjacks (less than 1-year-old) were caught in the wild and subsequently reared in offshore fish cages sited along the coast of the Zhaoan Gulf, Fujian, over two to three years. During this period, fish were fed on alternate days with small live or frozen fish. Gonadal tissues for histological analysis were collected in December 2019 and February 2021 when the fish reached 2–3 years of age. Hormonal treatments were performed in February and April 2021. Prior to dissection for tissue sampling or hormone injection, the fish were anaesthetised with clove oil. All animal procedures conducted in this study were performed in accordance with the principles of the Institutional Animal Care and Use Committee and were approved by the Animal Ethics Committee of the Institute of Hydrobiology, Chinese Academy of Sciences (Approval Code: IHB2019-0601, Approval Date: 1 June 2019).

### 2.2. Histological Processing

Three males and three females at each stage (2- or 3-year-old) were used for histological processing. Ovary and testis tissue samples were initially fixed in Bouin’s fluid (15 parts picric acid saturated aqueous solution, 5 parts 40% formaldehyde solutions A.R. *v*/*v*, 1 part glacial acetic acid) for 24–48 h, after which they were washed with 50% ethanol, dehydrated through an ethanol series, and embedded in paraffin. The embedded tissues were subsequently sectioned at 5 μm thickness and stained with haematoxylin and eosin (HE) for microscopic examination.

### 2.3. Body Weight, Body Length, and Gonadosomatic Index Determinations

In February 2021, 3-year-old sexually mature fish were used for body size determination. Body weight (BW), standard body length (BL), and gonad weight (GW). The gonadosomatic index (GSI) was calculated as GSI (%) = 100 × GW/BW. Statistical significance was determined using an independent sample *t*-test (*p* < 0.05).

### 2.4. Design and Synthesis of SdSNa and SdGnRHa

The amino acid sequences of the predicted greater amberjack Scg2 (XP_022616204.1) and GnRH1 (XP_022612160.1) proteins were determined based on searches in the NCBI database. The sequence of the greater amberjack Scg2 protein was compared with that of other species (from teleosts to mammals), based on which we identified a 34-amino acid conserved active peptide (denoted SdSNa) with the sequence TNENVEEKYTPQNLATLQSVFDELDKLTSTQTVH. Similarly, we compared the GnRH1 protein of the greater amberjack with that of other species, based on which we identified a decapeptide (denoted SdGnRH) with the sequence Gly-His-Trp-Ser-Tyr-Gly-Leu-Ser-Pro-Gly-NH2. To produce a synthetic SdGnRH agonist, we replaced Gly at position 1 with pyro-Glu, Gly at position 6 with dextrorotatory Trp, and Gly at position 10 with an ethylamide group (SdGnRHa: pGlu-His-Trp-Ser-Tyr-DTrp-Leu-Ser-Pro-NEt). Both SdSNa and SdGnRHa were synthesised by the GenScript Biotech Corporation (Nanjing, Jiangsu, China).

### 2.5. Hormone Measurements

Sexually mature females (3-year-old) were divided into three groups with six fish in each group, and injected with SdSNa/domperidone (DOM, a dopamine receptor antagonist), SdGnRHa/DOM, or saline (control group), respectively. Serum was collected 24, 48, 72, and 96 h post-injection. Serum hormones, including LH, follicle-stimulating hormone (FSH), thyroid-stimulating hormone (TSH), and oxytocin, were determined using dedicated enzyme-linked immunosorbent assay (ELISA) kits (Jiangsu Meimian Industrial Co., Ltd., Yancheng, Jiangsu, China) as previously described [27]. Measurements were performed according to the manufacturer’s instructions. Briefly, standards or samples were added to the appropriate microELISA strip plate wells and combined with a specific antibody. Horseradish peroxidase (HRP)-conjugated antibodies specific to hormones were added to each well. The free components were then washed away. TMB substrate solution was added to each well. Optical density (OD) was measured spectrophotometrically at a wavelength of 450 nm, and the hormone concentration in each sample was determined by comparing the sample OD to the standard curve. The means of six females in each treatment group were used to measure hormone levels, and the standard deviation of the mean (SD) was used to measure errors in these data.

### 2.6. Gene Expression Analysis

To compare gonadal gene expression between fish injected with normal saline and SdSNa/SdGnRHa/DOM mixture, we collected ovarian and testicular tissues (from 3-year-old sexually mature fish) for RNA-seq analysis. Fifteen sexually mature fish in each group were injected with normal saline or SdSNa/SdGnRHa/DOM in February 2021, and the gonads were dissected 48 h post-injection. For each treatment group, three ovaries or testes were randomly selected for RNA-seq analysis (*n* = 3 biological replicates). RNA-seq and data analyses were performed by Novogene Bioinformatics Technology (Tianjin, China). To map reads to the greater amberjack genome, we selected the TopHat2 algorithm, and fragments per kilobase million (FPKM) were used to normalise the expression data followed by expression level estimation and differential expression analyses using HTSeq and DESeq software (padj < 0.05). The genes (such as *wnt4* and *dazl*) associated with oocyte or sperm development in the ovary or testis were selected. The expression levels of these genes in hormone-treated fish were compared to those in control fish.

### 2.7. Spawning Induction Treatments

Wild-caught juvenile greater amberjacks were raised in seawater cages in the Zhaoan Gulf, China. Three-year-old sexually mature greater amberjacks (weighing more than 7 kg) were used for inducing spawning. Artificially induced spawning was performed during the reproductive season (February to April) in 2021. Fish that released milt could be reliably identified as males. The remaining fish were assumed to be females. Based on these criteria, we randomly selected five males and five females for each treatment group. Three groups (five males and five females in each group) were injected with different drug mixtures (Mixture 1: SdGnRHa 15 μg/kg fish, SdSN 15 μg/kg fish, DOM 3 mg/kg fish; Mixture 2: SdGnRHa 15 μg/kg fish, SdSN 15 μg/kg fish, DOM 3 mg/kg fish, human chorionic gonadotropin (HCG) 300 IU/kg; Mixture 3: HCG 300 IU/kg). The drug mixture was administered via a pectoral fin cavity injection. Following the injection, the fish in each group were placed in their respective cages for mating. At 2 h intervals, the surface water in cages was monitored for the presence of floating eggs, and the production of fertilised eggs was considered indicative of the success of the hormone cocktail treatment. Eggs were collected and incubated in seawater at 26 °C. The quality of the fertilised eggs was measured based on whether they developed into normal embryos. One thousand fertilised eggs were sampled to calculate hatchability.

### 2.8. Statistical Analyses

All data are presented as mean ± standard deviation (SD). Two-way ANOVA statistical analysis and Bonferroni post hoc tests were used to determine significant differences in serum hormone levels between drug-injected and control fish. Independent *t*-tests were used to detect significant differences in gene expression between hormone-treated and control fish. Differences were considered statistically significant at *p* < 0.05.

## 3. Results

### 3.1. Modification and Synthesis of SdSNa and SdGnRHa

**Figure 1:**
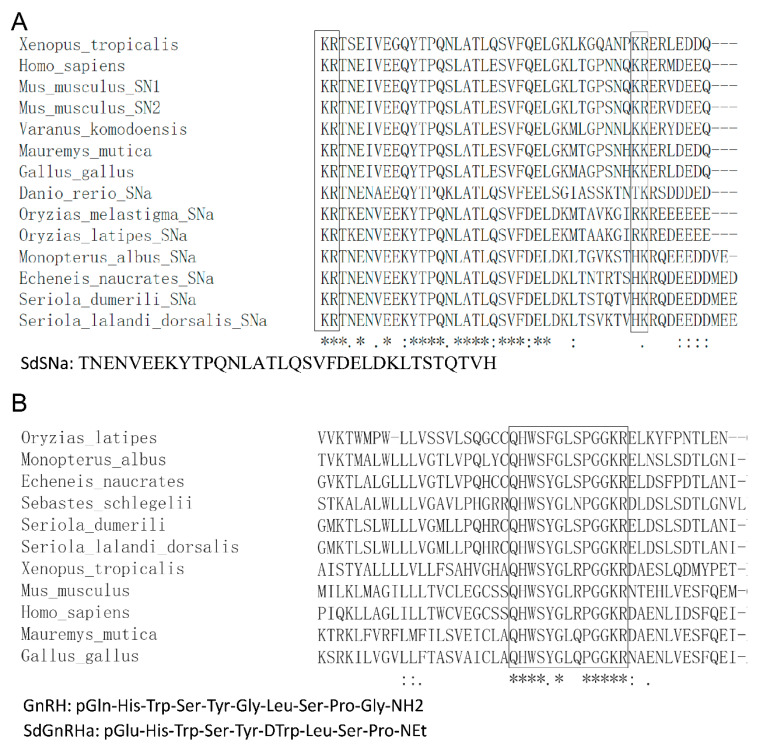
Comparative analysis of the secretoneurin active peptide (SNa) and gonadotropin-releasing hormone (GnRH) protein sequences of multiple species. (**A**) SNa consists of a conserved 34 amino acids active peptide. (**B**) GnRH consists of a conserved decapeptide. Accession IDs of secretogranin-2 proteins are listed in Supplemental Table S1 and accession IDs of gonadotropin-releasing hormone proteins are listed in Supplemental Table S2. The * indicates the most conserved amino acids.

Based on our searches of the NCBI database, we found genes encoding the greater amberjack SCG2, namely *scg2a* (Gene ID: 111232823) and *scg2b* (Gene ID: 111228946). Homology and phylogenetic analyses revealed that *scg2a* in greater amberjacks are closely related to SCG2 in mammals and birds. Multiple sequence alignment of the secretoneurin a (SNa) proteins of the greater amberjack and other fishes revealed the presence of conserved 34-amino acid peptides with potential monobasic cleavage sites (lysine and arginine) in both flanking sequences. Having identified this conserved sequence, we proceeded to synthesise the core peptide SdSNa, the amino acid sequence of which is shown in Figure 1A.

Homology analysis revealed that the GnRH1 core decapeptide identified in the greater amberjack is conserved among species of different taxonomic groups. The native decapeptide had the sequence Gln-His-Trp-Ser-Tyr-Gly-Leu-Ser-Pro-Gly-NH2. To prevent rapid degradation in the circulatory system, we modified the GnRH decapeptide sequence by substituting residues at positions 1, 6, and 10 with a pyro-Glu, a dextrorotatory (D) amino acid, and an ethylamide group, respectively, thereby yielding the agonist SdGnRHa (Figure 1B). The obtained SdSNa and SdGnRHa peptides were used to induce spawning in the greater amberjack.

### 3.2. Serum Hormone Levels after Treatment with SdSNa/DOM and SdGnRHa/DOM

**Figure 2:**
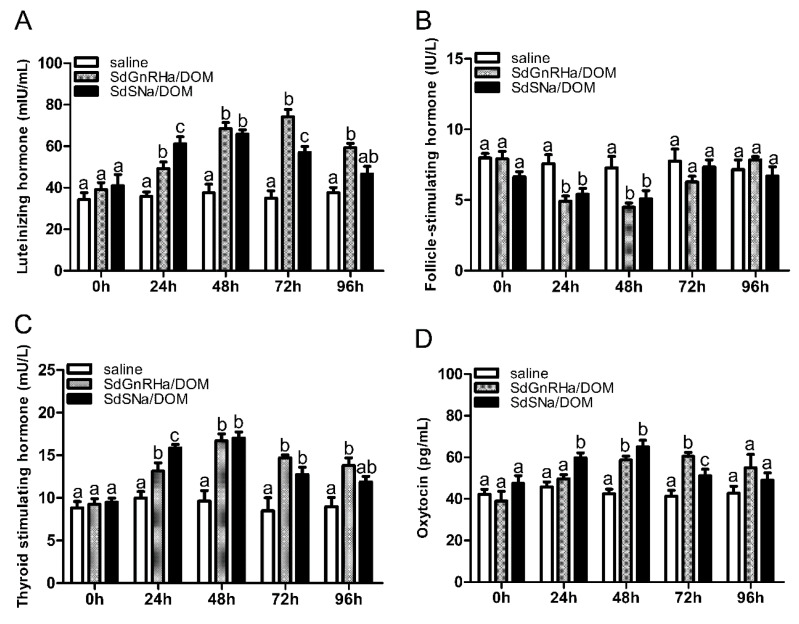
Serum hormone levels of *S. dumerili* after injection of SdSNa/DOM, SdGnRHa/DOM, and normal saline (*n* = 6). Serum levels of luteinizing hormone (**A**) and follicle-stimulating hormone (**B**) were increased and reduced, respectively, following injection with SdSNa/DOM or SdGnRHa/DOM. Serum levels of both thyroid-stimulating hormone (**C**) and oxytocin (**D**) were elevated in response to SdSNa/DOM and SdGnRHa/DOM treatment. The a, b, c indicates the indicate statistically significant differences, *p* < 0.05.

To ascertain the physiological effects of the synthetic peptides SdSNa and SdGnRHa, the serum levels of LH, FSH, TSH, and oxytocin were monitored after drug injection. We found that females injected with SdSNa/DOM were characterised by a significant elevation in serum LH at 24 h, which peaked at 48 h and subsequently declined. The levels remained significantly higher than those in the control group at 72 h and returned to levels comparable to those recorded in the control group at 96 h (Figure 2A). Similarly, in females injected with SdGnRHa/DOM, we detected significantly higher levels of LH at 48 h compared to those in the control fish, with levels peaking at 72 h, after which they declined. Notably, when measured 96 h post-injection, the levels of LH were still significantly higher than those in the control fish (Figure 2A). In contrast, we detected significant reductions in serum FSH levels at 24 and 48 h in both SdSNa/DOM- and SdGnRHa/DOM-injected fish, which subsequently returned to levels similar to those in the control fish at 72 h (Figure 2B). In addition to gonadotropins, we observed increases in serum TSH and oxytocin concentrations in response to injection with SdSNa/DOM and SdGnRHa/DOM, with the levels in the former being significantly higher than those in the control group at 24, 48, and 72 h post-injection. However, while the TSH concentrations in fish treated with SdSNa/DOM returned to levels similar to those recorded in the control group, the levels in the SdGnRHa/DOM-injected fish remained significantly higher at the end of monitoring (96 h) (Figure 2C). In contrast, the levels of oxytocin in the serum of SdSNa/DOM-injected fish were significantly higher at 24 h, peaking at 48 h, and subsequently returning to levels comparable to those in the control group at 72 h. However, the response was somewhat delayed in SdGnRHa/DOM-treated fish, showing significantly higher levels at 48 h, peaked at 72 h, and returning to control levels at 96 h (Figure 2D).

### 3.3. Gene Expression after Treatment with SdSNa/SdGnRHa/DOM

**Figure 3:**
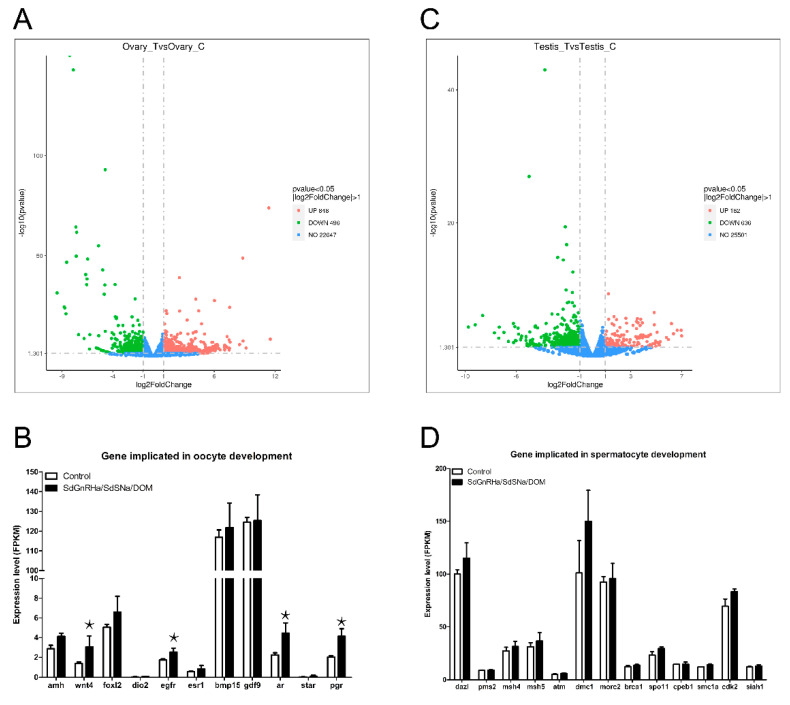
Transcriptome analysis of differentially expressed genes in the gonads of SdSNa/SdGnRHa/DOM injected *S. dumerili* (*n* = 3). (**A**) An MA plot showing the significantly downregulated (in green) and upregulated (in red) genes in the ovaries of injected females. (**B**) Genes implicated in oocyte development were upregulated after injection. (**C**) An MA plot showing significantly altered genes in the testes of injected males. (**D**) Genes implicated in spermatocyte development that were upregulated in the SdSNa/SdGnRHa/DOM injected *S. dumerili*. The ＊ indicates statistically significant differences, *p* < 0.05.

Transcriptomic analysis of gonadal gene expression in SdSNa/SdGnRHa/DOM-injected fish revealed that 848 genes were up-regulated in the ovaries of female fish, whereas 496 genes were down-regulated (Figure 3A). It is worth noting that several key regulatory factors of oocyte development (including *wnt4* and *egfr*) were among the up-regulated genes. Similarly, the expression of the progesterone receptor (*pgr*) which is essential for oocyte maturation, was found to be significantly upregulated in the ovaries of treated fish (Figure 3B). We detected 162 up-regulated and 636 down-regulated genes in the testes of male fish in response to SdSNa/SdGnRHa/DOM injection (Figure 3C). Among the upregulated genes, factors associated with spermatogenesis and meiosis were identified (Figure 3D).

### 3.4. Gonadal Development and Sexual Maturation of Greater Amberjack

**Figure 4:**
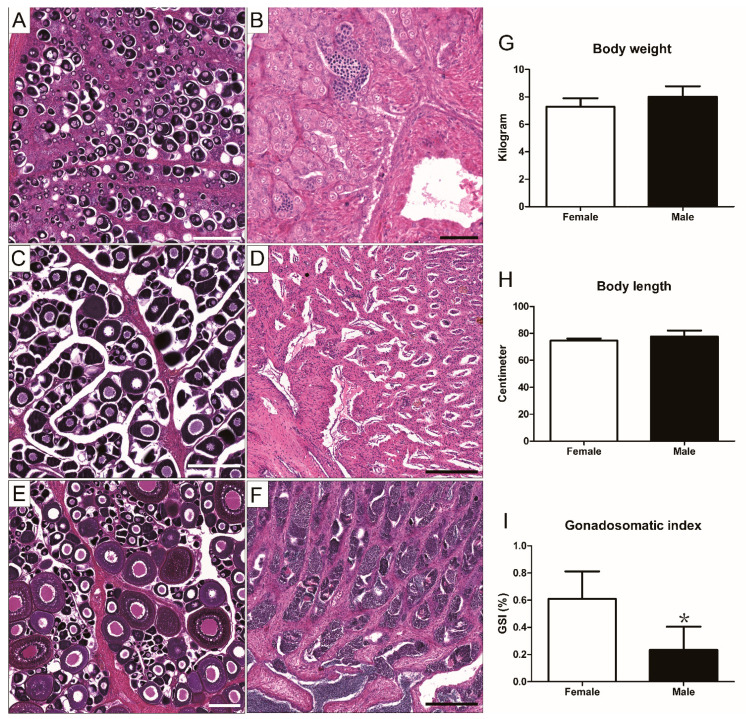
Micrographs of gonadal tissue sections sampled at three different growth stages from male and female *S. dumerili*, and the body weight, body length, and gonadosomatic index of sexually mature individuals were measured during the breeding season. (**A**) Oogonia and primary growth oocytes were detected in the ovaries of 2-year-old fish. (**B**) Spermatogonia and a few meiotic-stage spermatocytes were detected in the testes of 2-year-old males. (**C**) Perinucleolar oocytes as the most advanced stage in the ovaries of 3-year-old females during the non-reproductive season. (**D**) Although seminiferous ducts were observed at this time, no sperm was detected. (**E**) Vitellogenic oocytes in the ovaries of 3-year-old females in February. (**F**) On entering the reproductive season, the seminiferous ducts of male fish were filled with mature sperm. No significant differences were detected in the body weight (**G**) or body length (**H**) of male and female fish. The gonadosomatic index (**I**) of females was notably higher than that of males. Scale bar: 200 μm (**A**,**C**–**F**), 50 μm (**B**). The ＊ indicates statistically significant differences, *p* < 0.05.

A correlation between gonadal development and age in the captive-reared greater amberjack was established based on histological analysis, which revealed that both males and females were still immature at 2 years of age. In the ovaries of females, oogonia and primary growth oocytes accumulated around the gonadal epithelium (Figure 4A), whereas in the male testes, almost all germ cells were at the spermatogonial stage. Although sporadic meiotic germ cells were observed at this time, no mature sperms were detected (Figure 4B). These observations indicate that males and females undergo sexual maturation at a stage of development after reaching 2 years of age, which we observed in a 3-year-old individual. During the non-reproductive season, oocytes were in the primary stage of growth. Almost no oogonia were observed in the ovaries (Figure 4C). In the testes, although we observed seminiferous ducts, no sperm was detected during the non-reproductive period, and relatively few spermatogonial cells were detected in the vicinity of the gonadal epithelium (Figure 4D).

Gonad morphological analysis revealed that oocyte vitellogenesis and spermatogenesis occurred actively in the ovary or testis in February in 3-year-old greater amberjack raised on the coast of southern China. Oocytes at different developmental stages (perinucleolus stage, primary yolk globule stage, full growth stage) were observed in the ovaries (Figure 4E). In males, we detected evidence of vigorous spermatogenesis, with seminiferous ducts filled with masses of mature sperm (Figure 4F). The average body weight and length of sexually mature males were 8.02 ± 0.25 kg and 77.7 ± 1.48 cm, respectively, which did not differ significantly from that recorded for sexually mature females (7.28 ± 0.31 kg and 74.5 ± 0.87 cm) (Figure 4G,H). However, the gonadosomatic index was significantly higher in females than in males (Figure 4I).

### 3.5. Spawning-Induction Treatments

**Figure 5:**
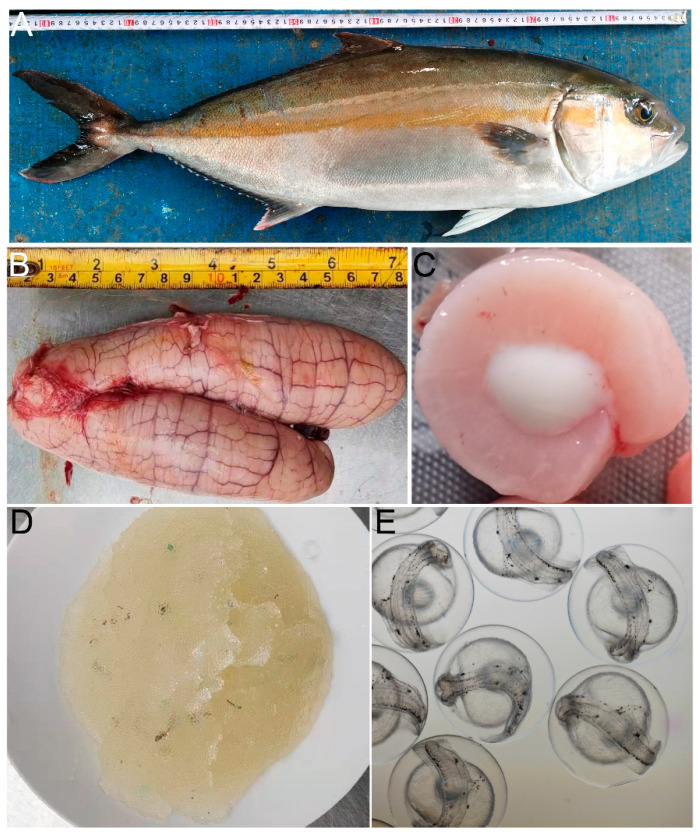
Hormonal treatments were used to induce the spawning of *S. dumerili*. (**A**) Body size of 3-year-old sexually mature greater amberjack. The ovaries (**B**) and testes (**C**) were sampled to assess the developmental and mature stages. (**D**) The fertilized eggs were collected and weighed. (**E**) The development of fertilized eggs into embryos.

A 3-year-old sexually mature greater amberjack was used in the spawning-induction treatments (Figure 5A). In late February, we attempted to induce spawning by injecting hormone cocktails, but this failed. This could be because the gonads were not sufficiently mature. We then examined gonadal maturation in April, when the ovaries were repleted with fully grown oocytes (Figure 5B) and the testes contained a large number of mature spermatozoa (Figure 5C). In April 2021, three groups of fish were injected with different drug mixtures (SdGnRHa/SdSN/DOM, SdGnRHa/SdSN/DOM/HCG, and HCG respectively). No spawning was detected during the subsequent 48 h in the groups injected with SdGnRHa/SdSN/DOM or HCG. Spawning (as indicated by the presence of floating fertilised eggs) was detected between 36 and 40 h in the group injected with SdGnRHa/SdSN/DOM/HCG. Fertilised eggs were collected and they weighed 0.8 kg (Figure 5D). Based on the number of fertilised eggs obtained, we assume that at least three of the five females had laid eggs. Fertilised eggs were incubated indoors at 26 °C, with developed embryos hatching after 30 h. The embryo hatching rate was 80% (Figure 5E).

## 4. Discussion

In this study, we used the active peptide of SN, derived from the precursor protein SCG2, as a stimulator to induce spawning in fish. Of the two *scg2* paralogs of SCG2 (*scg2a* and *scg2b*) found in the greater amberjack, homology and phylogenetic analyses revealed that *scg2a* is closer to SCG2 in mammals and birds. Therefore, we speculate that the physiological function of SNa may be more conserved and that it plays a role in regulating gonadotropin. Subsequent multiple sequence alignment of precursor SCG2 proteins in the greater amberjack and other fishes revealed that SCG2s of different species contain conserved sequences of 34 amino acids (SN) characterised by potential monobasic cleavage sites, lysine, and arginine, in both flanking sequences. Using a combination of reverse-phase HPLC and mass spectrometry analysis, the 34-amino acid-free peptide has previously been isolated from the whole brains of goldfish [23], which suggests that in greater amberjack, SCG2 hydrolysis may yield a 34-amino acid SN that functions as a physiologically active form. Based on this assumption, we synthesised peptide SdSNa to examine its effects on promoting pituitary hormone release. Pioneering research in goldfish has revealed that the GABA-transaminase inhibitor γ-vinyl-GABA upregulates an SCG2-like pituitary transcript that promotes LH release in vivo [21]. SN immunoreactivity has been detected in prolactin cells in the rostral pars distalis of the anterior pituitary gland, and the effect of SN was found to be stronger than that of GnRH in promoting LH release from dispersed goldfish pituitary cells [23]. Furthermore, preincubation with a specific goldfish SNa polyclonal antibody has been found to partially block the stimulatory effect of GnRH3 on LH release. This is the basis on which it has been hypothesized that PRL cells release SNa-like products that influence the activity of LH-releasing cells via a paracrine pathway [25]. It has also been proposed that the stimulatory effect of GnRH on pituitary gonadotrophins may be suppressed by the potent inhibitory activity of dopamine in some teleosts [28,29]. Thus, to counter this inhibitory effect, a dopamine antagonist (such as DOM) is routinely co-injected with GnRH when these fish performed artificial inducing spawn. Intraperitoneal injection of SNa in combination with a dopamine receptor blocker has indicated that SN can robustly stimulate anterior pituitary function in vivo [21,22]. Consistent with this, we demonstrated that co-injection of greater amberjack with SdSNa and DOM promoted a notably strong release of pituitary hormones. Moreover, we detected significant increases in the levels of LH, TSH, and oxytocin in the sera of fish receiving combined SdSNa/DOM treatment. These observations not only verified the role of SN in stimulating gonadotropin release in the greater amberjack but also indicated that SNa can promote the release of multiple pituitary hormones. Similarly, SNa induces a significant elevation in the mRNA levels of hypothalamic GnRH, pituitary LHβ, and FSHβ in the orange-spotted grouper *Epinephelus coioides* [30]. Furthermore, gene knockout analyses in zebrafish have provided evidence that SCG2 plays a pivotal role in a range of reproductive processes, including sexual behaviour, brain and pituitary gene expression, and spawning [26]. Collectively, the findings of these studies indicate that SNa regulation of gonadotropin release is a conserved process in fish, and consequently, the SN can be considered an ideal target for the development of fish spawn stimulators. This is particularly relevant in the case of cultured fish, in which successful spawning is difficult to obtain.

GnRHas have been extensively used as components of spawning induction treatments. In the early study, GnRHa implants were used to promote gamete maturation and spawning in cultured greater amberjack. The results showed that single implantation induced a small number of oocyte maturation and promoted limited ovulation [31]. Later, consecutive GnRHa injections were used to induce spawning of greater amberjack reared in tanks, with 22 spawning events being recorded in response to a total of 15 inductions in four males and two females [32]. The effects of a rotation injection and implantation of GnRHa on inducing spawn were compared in captive greater amberjack. The egg quality was significantly higher in injected fish than in implanted fish [33]. Recently, the GnRHa implants were performed for greater amberjack reared in tanks and sea cages. Females reared in sea cages were eligible for spawning induction, while females reared in tanks showed almost 0% fertilization success [34]. However, procedures entailing multiple embedding or injections are not only complex but can also render fish more susceptible to infection and death. To overcome these problems, we designed GnRH analogues based on sequences of the native GnRH core peptide of the greater amberjack. Substitutions at positions 6 and 10 in the GnRH decapeptide with a dextrorotatory (D) amino acid and an ethylamide group, respectively, have been performed to produce super-active agonists that are resistant to enzymatic degradation [35,36,37]. The synthetic SdGnRHa peptide thus obtained was more likely to bind to its receptor, thereby playing a normal physiological role in promoting gonadotropin release. Our observations of significantly elevated levels of LH, TSH, and oxytocin, but not FSH, in the sera of fish injected with SdGnRHa/DOM, suggest that the peptide SdGnRHa synthesised in this study can play a functional role in promoting pituitary hormone release in the greater amberjack. A previous study also showed that GnRHa implants in female greater amberjack elevated LH, but not FSH plasma levels within 42 h of administration [38]. Accordingly, we established that the administration of SdGnRHa can contribute to an integrated stimulation of reproductive processes by directly or indirectly inducing the release of other hormones involved in oocyte maturation, such as a thyroid-stimulating hormone. In a recent study, commercial GnRHa (Des-Gly^10^, D-Ala^6^-Pro-NEth^9^-mGnRHa) was administered to controlled-release implants to induce spawning in a wild-caught stock of greater amberjack reared in sea cages [39]. Further studies are needed to compare the effect of our SdGnRHa with commercial GnRHa using fish in the state.

In this study, the comprehensive physiological effects of SdSNa/SdGnRHa/DOM injections were explored by analysing changes in the transcriptome of gonadal tissues. The expression of genes associated with oocyte development and maturation in ovaries increased after drug injection. *wnt4* has been demonstrated to play important roles in ovarian development in teleost such as *Acanthopagrus schlegelii* [40]. Our previous study also found that EGFR signalling is critical in zebrafish early folliculogenesis [41]. The level of the nuclear progestin receptor (*pgr*) is associated with ovulation and egg spawning [42]. In this study, the key regulatory factors of oocyte development (*wnt4*, *egfr*, and *pgr*) were upregulated when SdSNa/SdGnRHa/DOM was injected. This suggests that drug injection promotes the expression of genes critical for the development and maturation of oocytes in the ovaries. Although the number of upregulated genes in the testis was less than that of downregulated genes, the expression of spermatogenesis-related genes showed an upward trend. These results further explain the physiological function of SdSNa/SdGnRHa/DOM injection. In order to obtain a better effect, a combination of drugs was injected in this study. A more comprehensive study should be taken place to confirm the efficacy of each of the substances.

GnRH and SN produced in the hypothalamus act on the pituitary gland to stimulate the release of gonadotropic hormones (GtH), including FSH and LH. If pituitary LH stores are insufficient, the effects of GnRHa and SN injections can be significantly reduced. HCG has a high degree of structural homology with LH [43], which has been employed in spawning induction trials of many species in culture. The long half-life of HCG in circulation and its prolonged effect on gonadal maturation induces spermatogenesis and spermiation in Japanese eel [44]. In this study, the injection of SdGnRHa/SdSN/DOM indirectly regulated spawning by promoting the release of pituitary hormones such as LH. Pituitary hormone stores might be insufficient in the captive greater amberjack. The injection of the SdGnRHa/SdSN/DOM mixture did not produce enough LH and failed to induce spawning or requires a longer time. The addition of HCG to the injected drug could compensate for the deficiency of LH and play a more effective role. However, HCG injection alone was not sufficient to induce spawning. Admittedly, it is not certain that the combination of hormones in this study is the best, which requires further trial and adjustment.

In summary, we designed and synthesised specific bioactive peptides, SdSNa and SdGnRHa, from greater amberjack. SdSNa and SdGnRHa were used as the main components to prepare a compound hormone cocktail (SdGnRHa/SdSN/DOM/HCG). Administration of this cocktail was found to be effective in inducing mating and spawning of male and female amberjacks. It is assumed that this approach will be equally applicable for inducing spawning in other fish that are difficult to breed in an aquaculture environment after suitable modification.

## Data Availability

Access to the data presented in this study is available in the Materials and Mehtods section and in the Supplementary Material section.

## Supplementary Materials

The following supporting information can be downloaded at: https://www.mdpi.com/article/10.3390/life12091457/s1, Table S1: GeneBank IDs of secretogranin-2 protein sequences used for comparative homology analysis, Table S2: GeneBank IDs of gonadotropin-releasing hormone protein sequences used in this study.
