# Supplementary material for: Effects of Secretoneurin and Gonadotropin-Releasing Hormone Agonist on the Spawning of Captive Greater Amberjack (Seriola dumerili)
Source: Life (Basel). 2022 Sep 19;12(9):1457. doi: 10.3390/life12091457 (PMC9505948; doi:10.3390/life12091457)
Supplement: Supplementary file 1 [file life-12-01457-s001.zip › life-1890885-supplementary.pdf]

Supplemental Table S1

GeneBank IDs of secretogranin-2 protein sequences used for comparative homology analysis.

**Species name**	**Accession number**
Xenopus tropicalis	NP_001072311.1
Homo sapiens	NP_003460.2
Mus musculus (isoform 1)	NP_033155.1
Mus musculus (isoform 2)	NP_001297609.1
Varanus komodoensis	XP_044288914.1
Mauremys mutica	XP_044885074.1
Gallus gallus	XP_040535214.1
Danio rerio	XP_009290228.1
Oryzias melastigma	XP_024133635.1
Oryzias latipes	XP_004075761.1
Monopterus albus	XP_020441343.1
Echeneis naucrates	XP_029374128.1
Seriola dumerili	XP_022616204.1
Seriola lalandi dorsalis	XP_023284183.1

Supplemental Table S2

GeneBank IDs of gonadotropin-releasing hormone protein sequences used in this study.

**Species name**	**Accession number**
Oryzias latipes	NP_001098169.1
Monopterus albus	AAW51121.1
Echeneis naucrates	XP_029365716.1
Sebastes schlegelii	AFS52222.1
Seriola dumerili	XP_022612160.1
Seriola lalandi dorsalis	XP_023249933.1
Xenopus tropicalis	XP_031753457.1
Mus musculus	NP_032171.1
Homo sapiens	NP_000816.4
Mauremys mutica	XP_044860640.1
Gallus gallus	NP_001074346.1
